# Supplementary material for: Experimental evidence for delayed post-conflict management behaviour in wild dwarf mongooses
Source: eLife. 2021 Nov 2;10:e69196. doi: 10.7554/eLife.69196 (PMC8562999; doi:10.7554/eLife.69196)
Supplement: Transparent reporting form [file elife-69196-transrepform1.docx]

***eLife’s* transparent reporting form**

We encourage authors to provide detailed information *within their submission* to facilitate the interpretation and replication of experiments. Authors can upload supporting documentation to indicate the use of appropriate reporting guidelines for health-related research (see [EQUATOR Network](http://www.equator-network.org/%20)), life science research (see the [BioSharing Information Resource](https://biosharing.org/)), or the [ARRIVE guidelines](http://www.plosbiology.org/article/info:doi/10.1371/journal.pbio.1000412) for reporting work involving animal research. Where applicable, authors should refer to any relevant reporting standards documents in this form.

If you have any questions, please consult our Journal Policies and/or contact us: [editorial@elifesciences.org](mailto:editorial@elifesciences.org).

**Sample-size estimation**

- You should state whether an appropriate sample size was computed when the study was being designed
- You should state the statistical method of sample size computation and any required assumptions
- If no explicit power analysis was used, you should describe how you decided what sample (replicate) size (number) to use

Please outline where this information can be found within the submission (e.g., sections or figure legends), or explain why this information doesn’t apply to your submission:

For Experiment 1, we used 17 focal individuals from all eight habituated groups in the population (see Materials and Methods 🡪 Experiment 1 protocol). A previous study on our population has shown that a minimum sample size of eight individuals is required to be able to detect a possible effect (Kern et al. 2016).

For the main experiment (Experiment 2), we used all our habituated mongoose groups (N=8) in the population (see Materials and Methods 🡪 Experiment 2 protocol). These groups are wild and have been habituated by members of the Dwarf Mongoose Research Project, which is very time consuming. This is the maximum number of groups we can work with for the size of our project.

**Replicates**

- You should report how often each experiment was performed
- You should include a definition of biological versus technical replication
- The data obtained should be provided and sufficient information should be provided to indicate the number of independent biological and/or technical replicates
- If you encountered any outliers, you should describe how these were handled
- Criteria for exclusion/inclusion of data should be clearly stated
- High-throughput sequence data should be uploaded before submission, with a private link for reviewers provided (these are available from both GEO and ArrayExpress)

Please outline where this information can be found within the submission (e.g., sections or figure legends), or explain why this information doesn’t apply to your submission:

For Experiment 1, each individual received two treatments in a repeated measures experimental design, with each treatment replicated 2–3 times per individual (see Materials and Methods 🡪 Experiment 1 protocol).

For the main experiment (Experiment 2), each group received two treatments in a repeated measures experimental design, with one replicate of each treatment (see Materials and Methods 🡪 Experiment 2 protocol).

**Statistical reporting**

- Statistical analysis methods should be described and justified
- Raw data should be presented in figures whenever informative to do so (typically when N per group is less than 10)
- For each experiment, you should identify the statistical tests used, exact values of N, definitions of center, methods of multiple test correction, and dispersion and precision measures (e.g., mean, median, SD, SEM, confidence intervals; and, for the major substantive results, a measure of effect size (e.g., Pearson's r, Cohen's d)
- Report exact p-values wherever possible alongside the summary statistics and 95% confidence intervals. These should be reported for all key questions and not only when the p-value is less than 0.05.

Please outline where this information can be found within the submission (e.g., sections or figure legends), or explain why this information doesn’t apply to your submission:

For details on the methods used for statistical analysis see ‘Experiment 1 protocol’ and ‘Experiment 2 protocol’ in the ‘Materials and Methods’ section. For more detailed information on the types of mixed models carried out for Experiment 2 see the legend of ‘Table 1’. For the output of our statistical analyses see the ‘Results’ section and ‘Table 1’.

(For large datasets, or papers with a very large number of statistical tests, you may upload a single table file with tests, Ns, etc., with reference to sections in the manuscript.)

**Group allocation**

- Indicate how samples were allocated into experimental groups (in the case of clinical studies, please specify allocation to treatment method); if randomization was used, please also state if restricted randomization was applied
- Indicate if masking was used during group allocation, data collection and/or data analysis

Please outline where this information can be found within the submission (e.g., sections or figure legends), or explain why this information doesn’t apply to your submission:

For Experiment 1, 17 randomly selected individuals received both treatments in a repeated measures design (see Results).

For the main experiment (Experiment 2), all groups got both treatments in a repeated measures design (see Results).

**Additional data files (“source data”)**

- We encourage you to upload relevant additional data files, such as numerical data that are represented as a graph in a figure, or as a summary table
- Where provided, these should be in the most useful format, and they can be uploaded as “Source data” files linked to a main figure or table
- Include model definition files including the full list of parameters used
- Include code used for data analysis (e.g., R, MatLab)
- Avoid stating that data files are “available upon request”

Please indicate the figures or tables for which source data files have been provided:

Source data files have been provided for our figures and statistical tests.
